# Supplementary material for: Identification of Molecular Subtypes and Prognostic Traits Based on Chromosomal Instability Phenotype-Related Genes in Lung Adenocarcinoma
Source: Cancers (Basel). 2024 Nov 13;16(22):3818. doi: 10.3390/cancers16223818 (PMC11592823; doi:10.3390/cancers16223818)
Supplement: Supplementary file 1 [file cancers-16-03818-s001.zip › Supplementary Figures.pdf]

Supplementary Figures

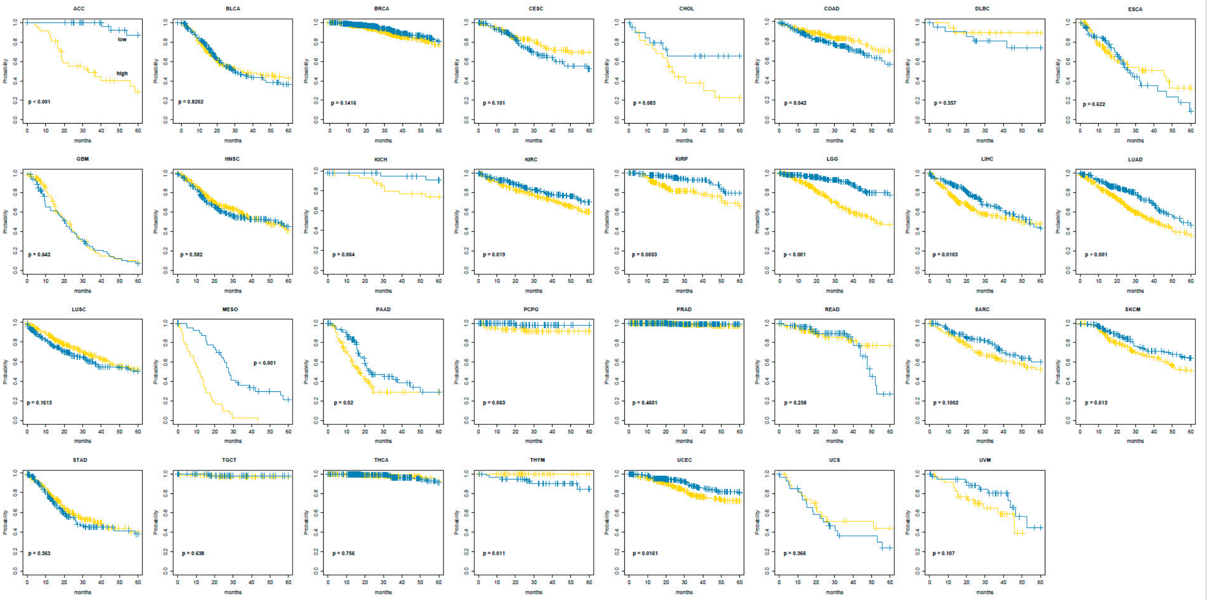

Figure S1. Kaplan-Meier curves of overall survival for patients based on the expression status (high expression > mean value; low expression ≤ mean value) of 24 LUAD-specific CIN-related genes (n = 24) across 31 TCGA cancer types. The p-value was calculated using a log-rank test. **Abbreviation**: ACC, Adrenocortical Carcinoma; BLCA, Bladder Urothelial Carcinoma; BRCA, Breast Invasive Carcinoma; CESC, Cervical Squamous Cell Carcinoma and Endocervical Adenocarcinoma; CHOL, Cholangiocarcinoma; COAD, Colon Adenocarcinoma; DLBC, Diffuse Large B-cell Lymphoma; ESCA, Esophageal Carcinoma; GBM, Glioblastoma Multiforme; HNSC, Head and Neck Squamous Cell Carcinoma; KICH, Kidney Chromophobe; KIRC, Kidney Renal Clear Cell Carcinoma; KIRP, Kidney Renal Papillary Cell Carcinoma; LGG, Lower Grade Glioma; LIHC, Liver Hepatocellular Carcinoma; LUAD, Lung Adenocarcinoma; LUSC, Lung Squamous Cell Carcinoma; MESO, Mesothelioma; PAAD, Pancreatic Adenocarcinoma; PCPG, Pheochromocytoma and Paraganglioma; PRAD, Prostate Adenocarcinoma; READ, Rectum Adenocarcinoma; SARC, Sarcoma; SKCM, Skin Cutaneous Melanoma; STAD, Stomach Adenocarcinoma; TGCT, Testicular Germ Cell Tumors; THCA, Thyroid Carcinoma; THYM, Thymoma; UCEC, Uterine Corpus Endometrial Carcinoma; UCS, Uterine Carcinosarcoma; UVM, Uveal Melanoma.

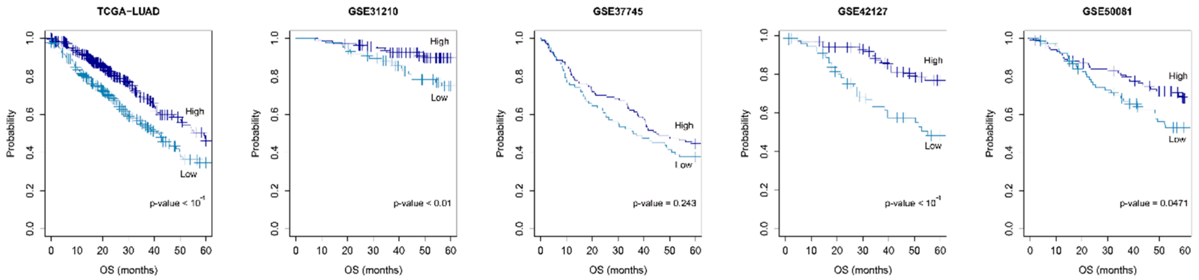

Figure S2. Kaplan-Meier curves of overall survival for patients based on the expression status (high expression > mean value; low expression ≤ mean value) of surfactant metabolism-related genes from the TCGA-LUAD and four independent cohorts. The *p*-value was calculated using a log-rank test.

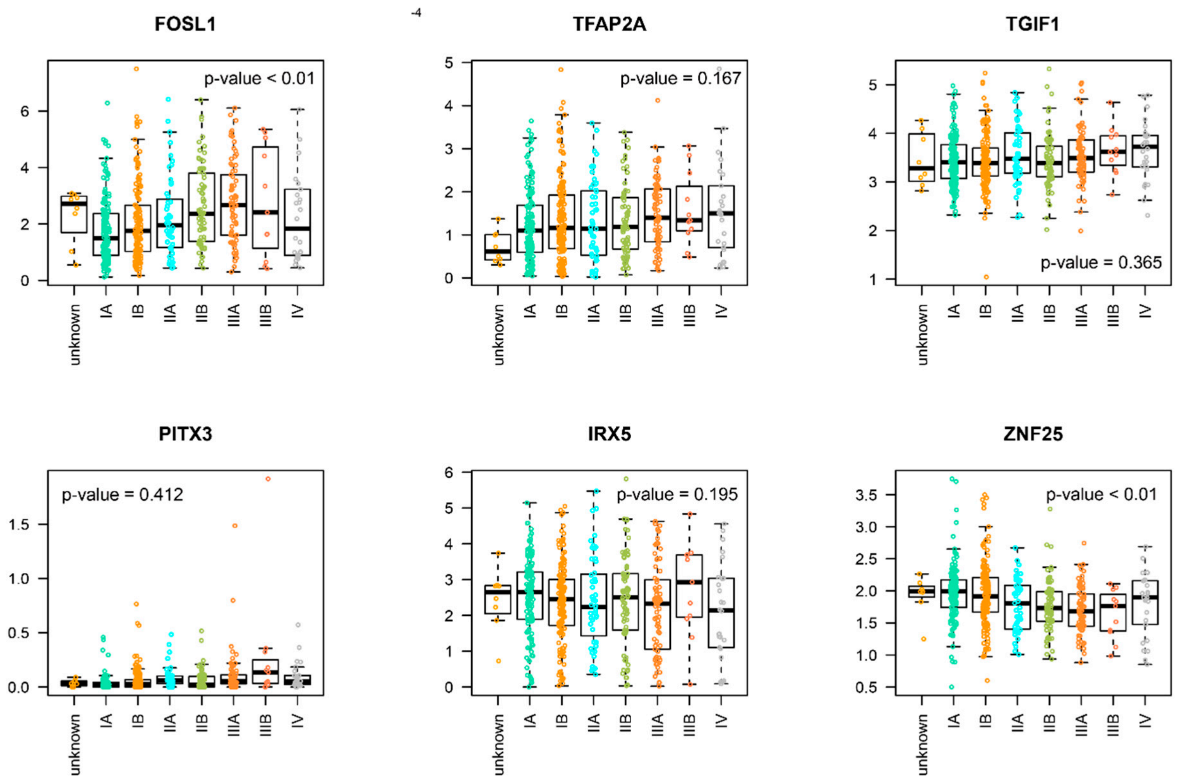

Figure S3. Boxplots show the expression levels of the indicated transcription factors (n = 6, see **Figure 6D**). The *p*-value was assessed using a one-way ANOVA.
